# Integrating camera imagery, crowdsourcing, and deep learning to improve high-frequency automated monitoring of snow at continental-to-global scales

**DOI:** 10.1371/journal.pone.0209649

**Published:** 2018-12-27

**Authors:** Margaret Kosmala, Koen Hufkens, Andrew D. Richardson

**Affiliations:** 1 Department of Organismic and Evolutionary Biology, Harvard University, Cambridge, Massachusetts, United States of America; 2 Unité mixte de recherche Interactions Sol Plante Atmosphère, Institut national de la recherche agronomique, Villenave d'Ornon, France; 3 School of Informatics, Computing and Cyber Systems, Northern Arizona University, Flagstaff, Arizona, United States of America; 4 Center for Ecosystem Science and Society, Northern Arizona University, Flagstaff, Arizona, United States of America; Griffith University, AUSTRALIA

## Abstract

Snow is important for local to global climate and surface hydrology, but spatial and temporal heterogeneity in the extent of snow cover make accurate, fine-scale mapping and monitoring of snow an enormous challenge. We took 184,453 daily near-surface images acquired by 133 automated cameras and processed them using crowdsourcing and deep learning to determine whether snow was present or absent in each image. We found that the crowdsourced data had an accuracy of 99.1% when compared with expert evaluation of the same imagery. We then used the image classification to train a deep convolutional neural network via transfer learning, with accuracies of 92% to 98%, depending on the image set and training method. The majority of neural network errors were due to snow that was present not being detected. We used the results of the neural networks to validate the presence or absence of snow inferred from the MODIS satellite sensor and obtained similar results to those from other validation studies. This method of using automated sensors, crowdsourcing, and deep learning in combination produced an accurate high temporal dataset of snow presence across a continent. It holds broad potential for real-time large-scale acquisition and processing of ecological and environmental data in support of monitoring, management, and research objectives.

## Introduction

Snow is a crucial component of Earth’s hydrology and affects climate at global scales. It affects the exchange of mass and energy between the land and atmosphere [1–4]. The magnitude and timing of snow melt have a huge influence on the seasonality of global hydrological cycles and input of freshwater to the world’s oceans [5–7]. And in the Arctic, the timing of snow melt affects the persistence of permafrost, with consequences for carbon release and global climate change [8,9].

Snow is also important at regional and local scales. In boreal and mountainous areas, the timing of snow melt affects spring vegetation phenology and related ecosystem functions such as pollination [10,11]. In many parts of the world, seasonal mountainous snow melt is a major source of surface water and groundwater recharge. It is important for power generation, irrigation, and as a source of drinking water for millions of people [12].

There is enormous spatial and temporal variation in rates of snow fall and snow melt, resulting in fine-scale heterogeneity in snow cover on the land surface, especially during early and late winter transition seasons. This variation is expected to change in complex ways over the next century [13], with repercussions for global and regional climate, hydrology, ecosystem functioning, and human societies [14].

Knowledge of snow cover is important at local, regional, and global scales, but accurate monitoring of snow is a challenge. During winter months, snow covers a large fraction of the global land area, and the cold and often remote locations where it falls make ground observations logistically challenging. Current approaches to snow monitoring include ground stations, airborne sensors, and satellite sensors. Each has its advantages and drawbacks. Automated ground stations can monitor snow at high temporal resolution but lack spatial extent. Airborne sensors can monitor snow across larger regions but are typically limited to specific seasons and have moderate temporal resolution. Satellite sensors can monitor snow globally and at high temporal resolution using measurements of reflected visible and infrared radiation, but are limited by cloud cover, forest cover, and a mismatch between fine-scale terrain heterogeneity and coarser satellite pixel size. Satellite sensors using passive microwave radiation avoid some of these limitations, but necessarily have coarser spatial resolution and the challenge of interpretation for snow packs of different grain size, density, and depth [13].

Networks of ground observations at high temporal and spatial resolution have the potential to increase the accuracy of snow monitoring products. Such measurements could be used as validation data for satellite sensors or as inputs to hybrid approaches that combine data from multiple types of sensors (e.g. [15,16]). SNOTEL provides an example of a dense snow-observing network [17]. However, SNOTEL’s coverage is limited to high-elevation mountainous areas in the western U.S., due to the expense of the instrumentation. Automated cameras have been used to track snow extent and depth for a couple decades. But until recently, they have been too expensive to deploy widely, resulting in studies that use no more than a handful of cameras to track snow in targeted areas [18–21].

Today, automated near-surface cameras are inexpensive and have been deployed to automatically record and transmit outdoor images for myriad reasons, including air quality monitoring, traffic monitoring, and vegetation monitoring. Tens of thousands of such cameras are already operational worldwide and present an opportunity to collect high frequency data about snow occurrence. However, automatically detecting snow in camera images is not easy. While the human eye can quickly determine whether snow is present or not in a given digital image, algorithms based on directly using the red, green, and blue color channels in the image (e.g. [19,22]) fail to consistently indicate snow presence or absence across a network of many cameras. Difficulties in accurate automated detection include differences in light levels and color balance among cameras, heterogeneous fields of view, and white objects in some cameras’ fields of view.

Recent advances in machine learning may offer a solution. A neural network technique known as “deep learning” has been shown to reach human-level accuracy in some computer vision tasks, such as facial recognition [23,24]. Deep learning shows promise in addressing data processing challenges in ecological and environmental fields. In the last few years, deep learning has been applied to the task of land cover classification from both satellite images [25–27] and crowd-tagged ground-based photography [28,29]. Specifically for snow, deep learning has been used in conjunction with support vector machines to classify snow in unlabeled Flickr images with 71.7% accuracy [30] and hand-labeled Flickr images with 80.5% accuracy [31]. One advantage of deep learning is that is can accommodate heterogeneous data, allowing for data processing of “found” data that can be analyzed for purposes other than for which it was originally gathered.

However, deep learning requires a large labeled dataset on which to train the identification algorithm. This presents a major challenge in applying such methods to real-world problems beyond the computer science discipline. For example, labeling tens or hundreds of thousands of images, even if the classification was simple e.g. “image has snow” or “image has no snow”, would be a time-consuming burden for a small research team. Updating these classifications in real time would add further challenge. Crowdsourcing, in which many human participants each perform a relatively small number of tasks, can make this challenge tractable. While this approach is well-known in computer science (e.g. Mechanical Turk [32]), it is not commonly used in many other disciplines. Integrating crowdsourcing and deep learning to answer ecological and environmental research questions is new within the past couple years.

We investigated the use of automated near-surface cameras as “snow detectors”, using crowdsourcing to develop a labeled dataset to which we then applied deep learning techniques. Our overarching question was, “Can deep learning be used to accurately detect snow in digital camera images?” The images we used came from the PhenoCam network, which is currently comprised of nearly 500 cameras (predominantly in North America) for vegetation monitoring [33]. We used crowdsourcing to determine the presence or absence of snow in nearly 185,000 images from 133 camera sites that were available to us at the time. We then trained a deep learning model on the image labels to automate the detection of snow in camera images. We tested the trained model using classic cross-validation methods. Finally, we compared our results with estimates of snow cover derived from satellite remote sensing (MODIS snow fractional cover product) as an example of how these data might be put to practical use.

## Methods

**Fig 1:**
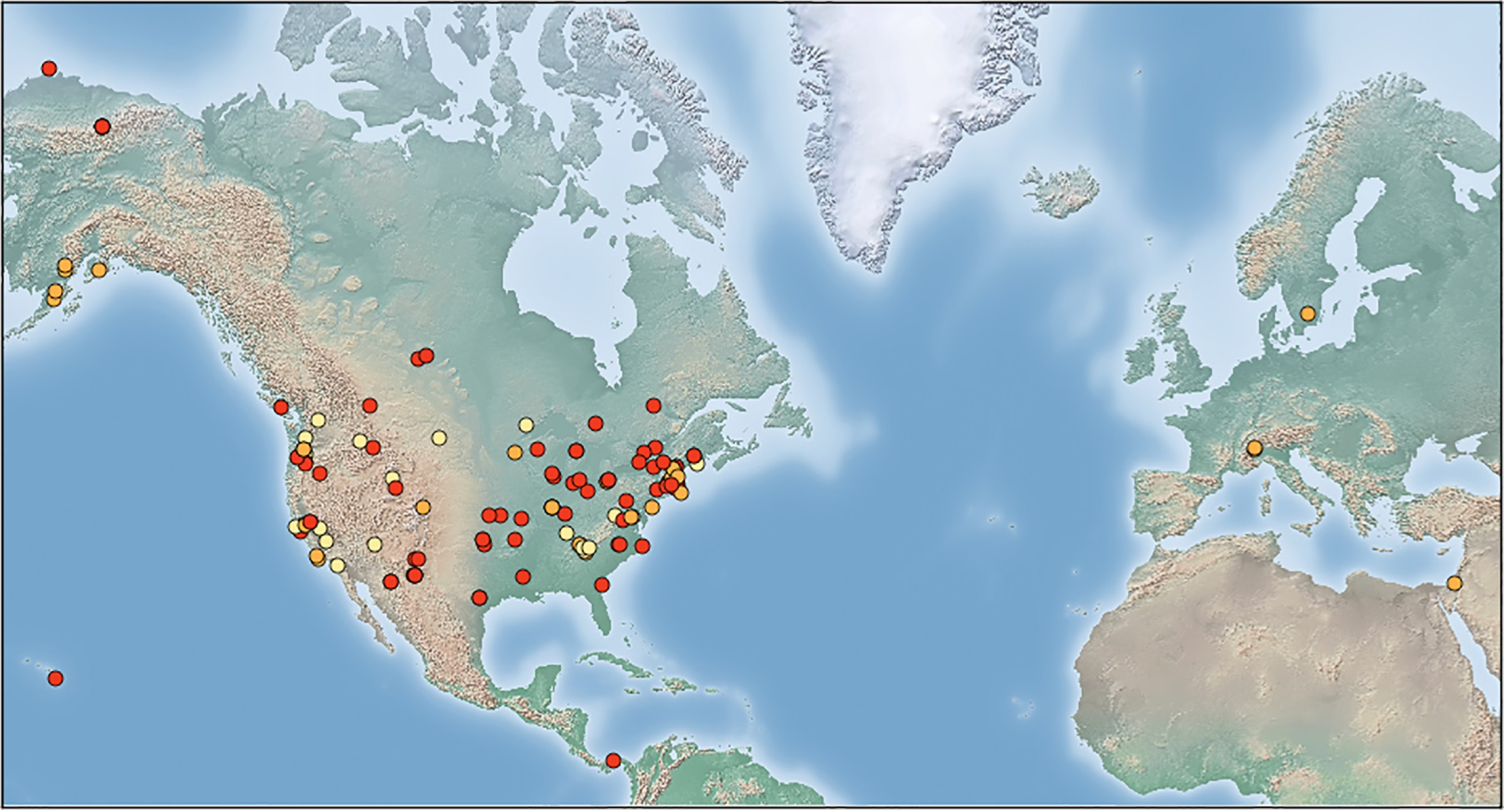
Location of PhenoCam camera sites used in the present analysis. Red: Type I sites; orange: Type II sites; yellow: Type III sites. Made with Natural Earth: free vector and raster map data @ naturalearthdata.com.

We used images collected by the PhenoCam network (http://phenocam.sr.unh.edu [33,34]), an expanding network of near-surface automatic cameras (Fig 1). At each site, cameras are mounted on towers, buildings, or other structures and take pictures across a landscape every half hour during the daytime, year-round. Sites are divided into Type I, Type II and Type III [33]. Type I sites use a standard camera and follow a protocol as to camera view and fixed white balance. Type II sites do not use the standard camera but use the Type I protocols. Type III sites are those that have imagery publicly available online and have been judged to meet the aims of the PhenoCam network, but do not necessarily use the standard camera or PhenoCam protocols.

We used imagery from the 133 PhenoCam sites that existed by 2014 (S1 Table). These sites encompass a wide range of North American ecosystem types, including deciduous broadleaf forest, evergreen coniferous forest, deciduous coniferous forest, mixed forest, savanna, shrubland, grassland, tundra, and wetland. Additionally, a wide range of human uses of the land are represented, including agricultural sites, urban and suburban sites, research sites, and protected natural sites. At each site, we used imagery from the start of collection until December 31, 2014. For each day of collection, we selected the image closest to noon local time to be representative of the snow presence or absence for that day. A total of 184,453 such midday images across all sites comprised the total imagery data set.

### 1. Crowdsourced classification

We contracted the company CrowdMobile to provide snow labels for the images. Each image was manually classified by three independent participants on Android-based mobile devices using the CrowdMobile crowdsourcing platform Knowxel [35,36]. Participants classified images as having snow, not having snow, or having poor quality such that it is not possible to distinguish whether snow is present or absent (‘bad image’). For images with trees and snow, participants also indicated whether snow was visible on the trees or whether it was only visible on the ground. We received from CrowdMobile each individual classification and consider the ‘crowd consensus’ classification for each image to be the classification that was chosen by at least two of the three participants (S2 Table). A crowd consensus could not be calculated for three of the images (<0.002% of the total), and these images were manually classified by the authors. We received no information about the participants from CrowdMobile.

To assess the accuracy of the crowd consensus, crowd consensus classifications were compared with a ‘gold standard’ set of classifications. From the total imagery data set, 2013 images (1% of the total) were randomly selected and classified by PhenoCam scientists using the same Knowxel platform employed by the participants. Each image was independently evaluated by three scientists, and images without unanimous consensus among scientists were reviewed and discussed by the authors to reach a definitive gold standard classification (S3 Table).

### 2. Deep learning classification

We used a deep convolutional neural network (CNN) to classify the presence or absence of snow in images. For CNN classification, we excluded images from the total dataset with a crowd classification of ‘bad image’; a total of 172,927 images remained for CNN classification. We used the Places365-VGG CNN [37] to classify each image. The Places dataset and corresponding CNNs result from forefront research on automatic scene classification. Our interest was to apply this technology to a pressing environmental research need, and so we chose the best-performing network according to the analyses performed by computer vision experts [37]. Places365-VGG is trained on the Places2 database and consists of a standard VGG architecture [38] of 16 convolutional layers. Its output is a prediction confidence score for each of 365 scene categories. We extracted the highest-scoring five categories (“top 5”) for each image and classified it as having snow if any of the categories of ‘iceberg’, ‘ice_skating_rink/outdoor’, ‘mountain_snowy’, ‘ski_resort’, ‘ski_slope’, or ‘snowfield’ were present, and not having snow otherwise.

We then used transfer learning to investigate whether Places365-VGG could achieve a higher accuracy than the simple “top 5” approach with existing categories. Transfer learning is a technique that uses an existing trained neural network to classify images or other data with a new set of labels. Accuracy is higher for the new labels when the old task and the new task are more similar [39]. Because the neural network already has a representation of the features in the images or data that then get mapped to labels, the raw network output can be used to redefine the mapping to labels. As a result, transfer learning is faster and less complicated than training a network from scratch and requires fewer training data.

We exchanged the 365 labels built into Places365-VGG for a pair of labels: ‘snow’ and ‘no snow’. We modified the source code for the Places2 CNN to extract the 4096 values of the nodes in the last fully-connected layer and used them as input to a support vector machine (SVM) with the output labels of ‘snow’ or ‘no snow’. We trained SVMs using the software LIBLINEAR (version 2.11, http://www.csie.ntu.edu.tw/~cjlin/liblinear [40]), with the solver set to ‘L2-regularized L2-loss support vector classification (primal)’ and with cost parameter = 0.0078 for all models. The cost parameter was chosen using LIBLINEAR’s cross-validation method for finding an optimal value. However, the models were not very sensitive to the cost parameter, with accuracies varying by just a half a percent or less for reasonable values. In general, LIBLINEAR solvers are not very sensitive to the cost parameter [40]. Final SVM weights are available in S4 Table.

We conducted two sets of 10-fold cross validation, training the SVM using 90% of the images and testing on the remaining 10% repeatedly until all images had been included exactly once in the testing group. For the first validation set, we split the images into training and testing groups randomly. For the second validation set, we kept images from the same site together when splitting into training and testing groups. The first method provides an accuracy estimate for classifying new images from existing sites. The second method estimates accuracy for classifying images from new sites.

There is considerable heterogeneity in the imagery from the PhenoCam network in terms of camera model, camera view, amount of vegetation, and camera configuration. Some camera sites are more similar to one another in terms of camera model, angle, orientation, and configuration, however, and it we did not know whether the CNNs would work better for prediction among a more homogenous subset of the full set of camera sites. To investigate this, we created two subsets of the CNN data set: a set that contains only images from Type I sites (82,698 images) and a set that contains images from Type I and Type II sites, but not Type III sites (126,908 images). We repeated the two sets of 10-fold cross validation on these two subsets of data to determine if camera configuration accounts for errors in CNN classification.

We also trained and tested the SVM on the data set and its two subsets in entirety. While these over-fitted models are not useful for prediction, they provide an upper bound for how well we can expect the transfer learning method to work for detecting snow using the Places2 CNN.

### 3. MODIS validation

For each image in the full dataset, we extracted the corresponding fractional snow cover value from MODIS products MOD10A1 and MYD10A1 [41]. These products are derived from radiance data from the Terra and Aqua satellites respectively and use a 500-meter resolution. Using values from both satellites increases the temporal coverage of MODIS data. We considered snow to have been detected if either the MOD10A1 or the MYD10A1 product had a fractional snow cover greater than zero. If neither product reported fractional snow cover corresponding to a given PhenoCam image (e.g. due to cloud cover), then we considered that image to have no MODIS information about snow and did not use it for the following analyses. A total of 96,151 images from the set of all images without a crowd consensus of ‘bad image’ (56%) had a MODIS classification of snow or no snow.

We calculated the rate of agreement between MODIS classifications and gold standard classifications, between MODIS classifications and crowd consensus classifications, and between MODIS classifications and the predictions of two CNN models. For the two CNN models, we chose a model that had a high accuracy and one that had a lower accuracy. This provided the range of possible agreements between MODIS classifications and those of the CNN models, without having to do comparisons with every CNN model. The higher accuracy model we used was the SVM trained and tested on Type I sites only, with a fully random split of the images (44,510 images with MODIS classifications). For the lower accuracy model, we chose the SVM trained and tested on all images, with images split by site.

We also examined the accuracy rates of MODIS classifications, as compared with the crowd consensus, for images containing trees and for those without trees. For those with trees, we further calculated accuracies based on whether snow was present on the trees or was only on the ground.

In comparing accuracies, we focused on true positive (snow is there and is classified as snow) and true negative (snow is absent and is classified as absent) rates, rather than overall accuracy because images without snow were much more frequent and accuracy rates for images with and without snow were very different. Only about 15% of the images with MODIS classifications had snow, and overall accuracy was considerably increased by the large number of true negatives.

## Results

### 1. Crowdsourced classification

Of all the images in the full data set, 6.2% were classified as ‘bad image’ by the Knowxel participants and were unusable for snow detection. The causes of these bad images were poor weather conditions, accumulation of water or ice on the lens, and camera malfunction. Of the remaining images, 72.8% were classified as not having snow; 4.0% were treeless images that had snow; 7.8% were images with snow on the trees and the ground; and 9.2% were images with snow on the ground, but not on the trees. We combined the latter three categories into a classification of ‘snow present’ for most analyses (38,727 images; 21.0%). The three classifications for each image agreed unanimously 94.0% of the time in classifying images as ‘snow’ or ‘no snow’, giving us high confidence in the overall quality of the individual Knowxel classifications.

When images with a crowd consensus of ‘bad image’ were removed from the gold standard dataset, the crowd consensus of ‘snow’ vs. ‘no snow’ had an accuracy of 99.1% compared to the gold standard (1797 images) for all sites and 99.4% for the datasets consisting of Type I and Type II sites (1352 images) and Type I sites only (924 images), respectively. (See S1 Appendix for confusion matrices.) These results indicate that the crowd consensus classifications are of excellent quality and can be used as reliably as an expert-annotated dataset.

### 2. Deep learning classification

Accuracy for the convolutional neural networks (CNNs) compared to the crowd consensus varied from 83.2% to 97.5%, depending on the specification of training and validation sets (Table 1 and S1 Appendix). Using the top-5 categories from Places365-VGG directly, accuracy was 83.2% for the full dataset. Transfer learning increased the accuracy to 97.0% when images were fully randomized between training and testing sets. The apparent upper bound was only slightly higher at 97.1%, suggesting that the CNNs with transfer learning would be excellent at predicting whether snow is present or not for new images from existing sites.

**Table 1:** Accuracies of convolutional neural networks (CNNs) as compared against crowd consensus classifications of ‘snow’ or ‘no snow’ for three datasets.

	Image dataset		
	Types I, II, III	Types I, II	Type I
SVM trained & validated on whole set	97.1%	97.4%	97.5%
SVM with 10-fold cross validation, random	97.0%	97.2%	97.2%
SVM with 10-fold cross validation, by site	91.6%	93.7%	93.5%
Places365-VGG using top-5 categories	83.2%	85.8%	85.5%

The three datasets: the full dataset, with images from Type I, II and III sites, and two subsets. Transfer learning was employed by replacing the classification step of Places365-VGG with a support vector machine (SVM) trained to distinguish between ‘snow’ and ‘no snow’.

Transfer leaning increased accuracy from 83.2% to 91.6% when images assigned to training and testing sets were split by site. This accuracy is lower than when training and testing sets were fully randomized and reflects the challenge for the CNNs in predicting whether snow is present or not at sites it has not yet seen. These new sites are outside the set of sites on which the CNN trained, and so the CNN is making inferences about images at these new sites based only on what it knows snow looks like at other sites. When a standard camera configuration was used for all the sites in the dataset (i.e. the subset datasets), the CNNs performed better on sites they had not yet seen; the difference in accuracy was 5.4% between the cross-validation models for the whole dataset, but just 3.5% and 3.7% for the two subsets.

CNNs trained on different sets of images almost always agreed on the snow classification for individual images. For the CNNs trained to predict new images from existing sites, the agreement rate was 99.4% and for those trained to predict images from new sites, the rate was 97.0%. For the small number of images where there was disagreement, the reason was usually because they were sites with small amounts of patchy snow. These images frequently also had non-unanimous consensus by participants.

Accuracy was always lowest for the full image dataset (Types I, II, and III), but by less than a few percentages points compared to the subset datasets. This suggests that a standard configuration across cameras has a small, but measurable impact on the ability of CNNs to learn to identify snow. Accuracies for the two subset datasets (Type I and II, Type I only) were similar, suggesting that choice of camera model does not affect the ability of CNNs to learn to identify snow if the cameras follow a standard configuration.

Sensitivity (true positive rate) for the CNNs with transfer learning was lower than specificity (true negative rate; Table 2). Specificity ranged from 94% to near perfect, whereas sensitivity for the most accurate CNN was 90.5% (S1 Appendix). This means that the CNNs fail to detect snow that is present (“missed snow”) much more often than they falsely detect snow that is not there (“phantom snow”). The overall CNN accuracies, therefore, reflect the high specificity, because images without snow are more frequent.

**Table 2:** Sensitivity (true positive rate) and specificity (true negative rate) for selected convolutional neural networks (CNNs) and datasets as compared against the crowd consensus.

	Types I, II, III			Type I		
	Sensitivity	Specificity	Accuracy	Sensitivity	Specificity	Accuracy
SVM trained & validated on whole set	89.3%	99.4%	97.1%	90.5%	99.6%	97.5%
SVM with 10-fold cross validation, by site	83.3%	94.0%	91.6%	86.0%	95.8%	93.5%

Accuracy rates are the same as those reported in Table 1.

**Fig 2:**
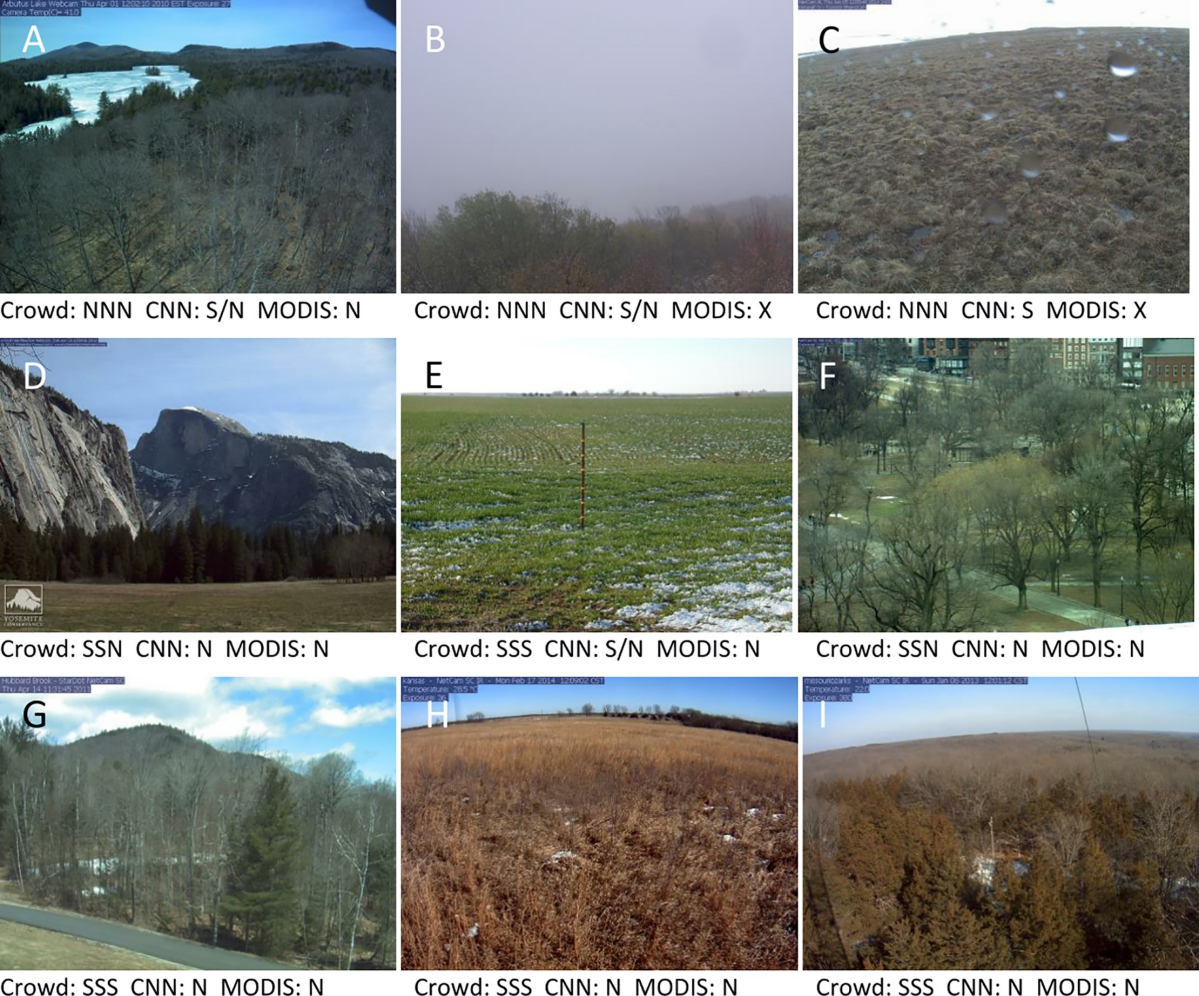
**Examples of false positive (A-C) and false negative (D-I) images.** Beneath each image are the labels provided by three participants (“Crowd”), convolutional neural networks (“CNN”), and a MODIS snow product. S = Snow, N = No snow, X = not available. False positives are due to (A) ice on lake, (B) fog, (C) precipitation on the lens. False negatives are due to (D) snow on distant mountains and (E-I) patchy snow.

We examined the reasons for misclassification by the CNNs. Snow was missed by the CNNs most often when there was little snow in an image and the snow was patchy (Fig 2). These “missed snow” images also appear to be difficult for humans to consistently classify. Of the “missed snow” images, 43% of them have non-unanimous classification, as compared with 6% for the whole dataset. Some of the errors were also due to human misclassification of images that do not have snow. There were few errors when snow was not present, and classification of images without snow was almost perfect in predicting new images from existing sites. The few errors were caused by misinterpretation of ice on lakes and rivers as snow, glare from wet pavement and metal equipment, fog and mist, and small amounts of precipitation on the camera lens (Fig 2).

### 3. MODIS validation

Overall accuracy of the MODIS snow product was high when validated against the gold standard, crowd consensus, and the most accurate CNN, and was only slightly lower when validated against the least accurate CNN (Table 3 and S1 Appendix). The majority of errors in all cases were images which had snow, but which MODIS missed. This is reflected in the sensitivity, which is substantially lower than the total accuracy in all cases (Table 3).

**Table 3:** Sensitivity (true positive rate), specificity (true negative rate), and accuracy of the MODIS fractional snow cover product as validated against four datasets based on ground time-lapse imagery from the PhenoCam network.

MODIS validated against:	Number of images	Sensitivity	Specificity	Accuracy
Gold standard	1044	81.7%	98.3%	95.5%
Crowd consensus	96,151	76.6%	97.1%	93.6%
SVM trained & validated on Type I images only	44,510	69.2%	95.0%	93.8%
SVM with 10-fold cross validation, by site, on all images	96,151	37.1%	94.9%	88.7%

The two CNNs are those with the highest and lowest accuracy respectively.

One recognized challenge in detecting snow with satellite sensors is when snow is present but lies beneath a tree canopy [42,43]. We found that sensitivity for sites with trees (75.4%) was somewhat lower than that for sites without trees (81.8%) when validated against the crowd consensus. More striking was that in cases when snow was visible on the tree foliage, sensitivity was much higher (88.9%) than when snow was only visible on the ground beneath the trees (67.9%). This affirms that visual obstruction of snow by vegetation causes the MODIS product to under-report snow [44–46].

Because the MODIS product and the CNNs both miss snow when it is present more often than they fail to detect snow when it is there, we checked to see if these two methods tend to make corresponding errors and whether a combined approach would increase sensitivity. We found that for all images with snow, the MODIS classification agreed with classifications from the most accurate and least accurate CNN 83.6% and 75.7% of the time respectively. We then created a combined classification by considering an image to have snow if either the MODIS classification or the CNN classification (or both) indicate snow. With this combined classification, we increased MODIS sensitivity from 76.6% (validated against the crowd consensus) to 91.4% for the most accurate CNN and to 89.4% for the least accurate CNN. The MODIS specificity drops somewhat for the combined classification system (to 95.4%/90.4% for the most/least accurate CNN) affecting overall accuracy, which increases for the most accurate CNN to 94.9%. The large increase in sensitivity and overall increase in accuracy indicate that MODIS and surface-based cameras together with deep learning image processing can be useful complementary methods of snow detection.

## Discussion

Using crowdsourcing and deep learning in conjunction with a network of automated near surface cameras provides an automated and accurate means by which the distribution of snow can be estimated, at high temporal frequency (daily), fine spatial resolution (10–100 m), and across a broad spatial extent (regional-to-continental). Crowdsourcing was as good as expert labeling for the task of identifying snow presence or absence in digital images and resulted in a high-quality human-labeled dataset. Using transfer learning and this dataset, we were able to train deep neural networks to determine the presence or absence of snow at 133 heterogeneous sites with up to 98% accuracy. This novel processing chain–involving automated sensors, crowdsourcing, and transfer learning of deep convolutional neural networks (CNNs)–has the capacity to accelerate the acquisition and processing of a wide range of data types for environmental monitoring and research.

### 1. Crowdsourced classification

For clear images, most of the time it was obvious whether or not snow is present to the human eye; 94% of clear images had unanimous agreement among the three classifying participants. Many of the non-unanimous classifications were cases in which there was very little snow, or when the snow was in the distant background (Fig 2). We had asked participants to count cases in which any snow was visible as ‘snow’. We recognize that if we had asked them to coarsely quantify the amount of snow (e.g. “no snow”, “some snow”, “mostly covered in snow”, “entirely covered in snow”), it may have resulted in a better training set. Images with little snow might then be more likely to be classified as “some snow” rather than “no snow” by the CNNs because the difference in amount of snow between adjacent classes would be smaller. Volunteer classifications were as accurate as expert classifications when combined into a consensus classification, demonstrating that simple tasks such as determining ‘snow’ versus ‘no snow’ can require as few three independent volunteer classifications for high accuracy. More difficult or complex tasks might require more classifications or other means to ensure data quality [47].

We crowdsourced labels to our entire image data set of 184,000+ images because we wanted a consistent and complete set of labels for a PhenoCam data product [33]. However, we could have been more efficient. Using temperature records from the various sites would have allowed us to quickly classify many images as having no snow because it is too warm, leaving far fewer for participants to classify. If we had only been creating a dataset for training CNNs, we could have been even more efficient and subsampled–by eliminating warm-weather images and using imagery from every-other day, for example, as consecutive days frequently look very similar [48].

### 2. Deep learning classification

While the overall accuracy of the CNNs was very high, the accuracy for images without snow was about ten percentage points higher than the accuracy for images with snow. The main reason that the CNNs missed snow was that for some images, snow was present in small amounts or was patchy (Fig 2). Presumably, this patchy snow did not match the gestalt of what a snow-covered landscape should look like, based on the majority of the training data. In many cases, though, images with patchy snow were correctly classified. These images with patchy snow were also the ones for which there was most likely to be disagreement among CNNs trained on different datasets, suggesting that they lie close to the hypersurface that separates classes within the state space of CNN parameters [49]. Different training sets are enough to shift that hypersurface slightly so that these “in between” images can fall on the “snow” side sometimes and on the “no snow” side other times. It is notable that the images with snow that were incorrectly classified by the CNNs, mostly due to patchy snow, had a much higher rate of disagreement among human participants than the average. This suggests that for some images, there may be a real ambiguity about whether snow is present or not. A more nuanced classification scheme could potentially address this issue.

For some applications, misclassifying patchy snow may not matter. PhenoCam images, for example, are used to track vegetation phenology, or “greenness,” over the seasons [33]. Broad swaths of snow that completely cover vegetation interfere with the greenness signal, but small amounts of snow do not. In tracking the emergence and flowering of alpine plants, a blanket of snow might signify a complete absence of these plants, whereas patchy snow may allow some plants to grow and bloom [50]. For other applications, it may be important to know whether small amounts of snow are present. Patchy snow can affect the exchange of energy and matter between land and atmosphere, for instance. Therefore, coupled land-atmosphere models may need to know not just whether snow is present, but also a measure of snow quantity [51].

**Fig 3:**
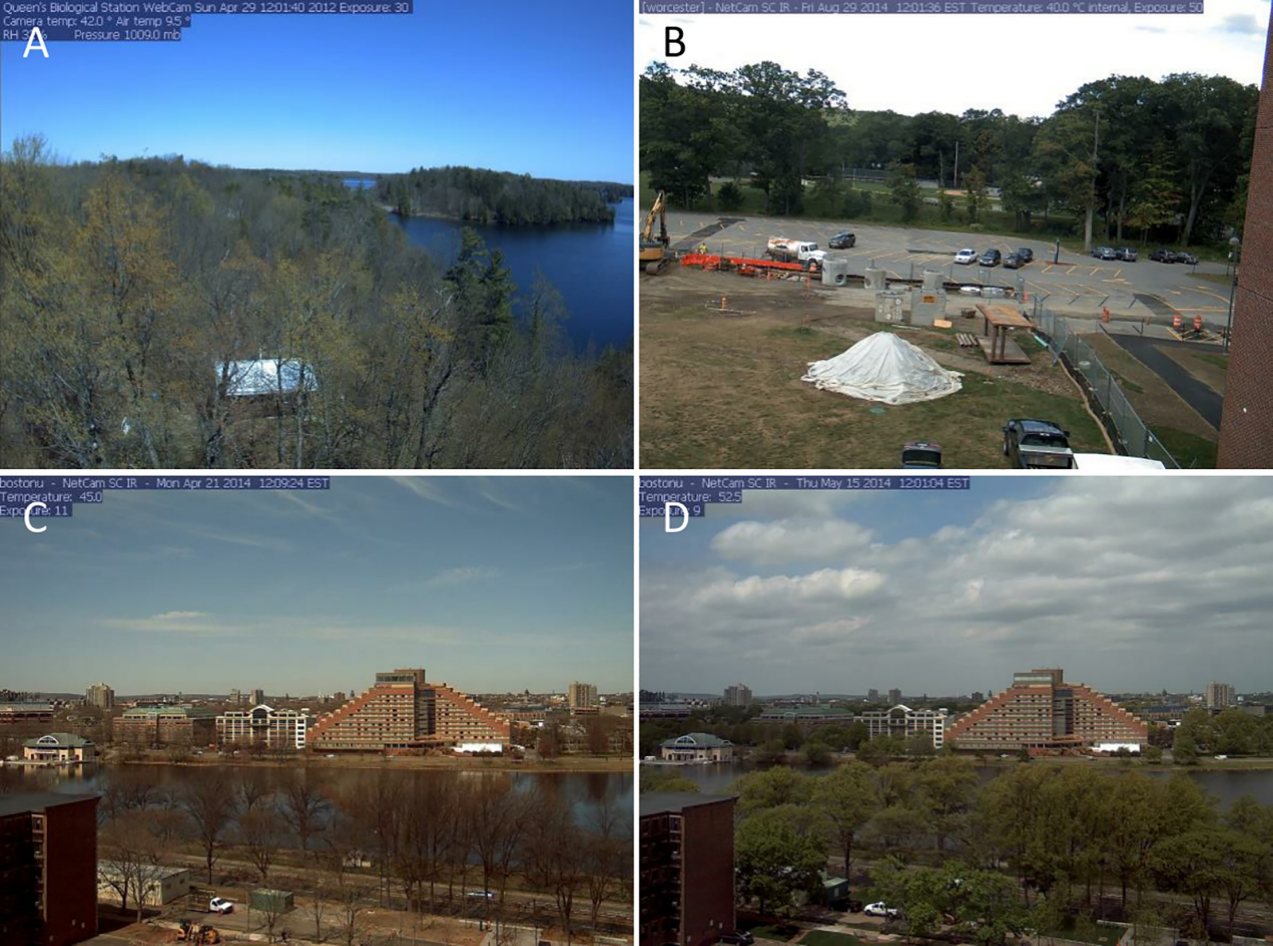
White object confusion. White objects can fool CNNs unfamiliar with a particular site into classifying images as “snow” when no snow is present (A-C). However, these CNNs frequently correctly classify very similar images correctly as having “no snow” (D).

One error type that occurred for CNNs trained to predict images from new sites, but not for those trained to predict new images from existing sites, was the presence of a sizable white object in the field of view causing the image to be classified as snow when no snow was present. These white objects could be permanent or temporary, and included white vehicles, buildings, and pieces of equipment (Fig 3). CNNs that were trained to predict snow in new images from existing sites were familiar with these sites and did not mistake the white objects for snow. However, the CNNs that were not trained on the sites with white objects were not always able to determine whether or not the white objects constituted snow. In computer vision, it is assumed that the test set is drawn from the same distribution of images as the training set [24]. When the test set constitutes a different distribution, errors are more likely to occur. However, because we used transfer learning, the CNNs should retain an underlying representation of white objects that are not snow from the original Places2 database [52]. In fact, the CNNs correctly classified some images with white objects, but no snow, as not having snow (Fig 3). However, some white objects look a lot like snow and it is easy to see how a CNN might interpret an object as snow if it was unfamiliar with that particular site (Fig 3).

There are several possible ways that overall accuracy of CNN snow detection might be improved. One idea is to add environmental data as input to the CNN along with each image. Geographic location, elevation, day of the year, and temperature provide much information about the likelihood of snow in an image. Deep learning has been shown to be effective at integrating different types of data, such as audio combined with video and imagery combined with text [53]. Combining satellite data with near-surface imagery as direct inputs to a CNN is another area for future research.

Another possibility is to take advantage of the auto-correlation in snow cover between subsequent days; if there was snow at a site one day, it is likely that there was snow there the next day, too. A prediction for the prior and/or subsequent image(s) could be used as input to a CNN along with a target image, potentially using a second round of CNN classification for images that have a different classification from surrounding images. Alternatively, the use of recurrent neural networks, which take sequential data as input, could be considered [24]. While recurrent neural networks have typically been used for language data like speech and text, insights gained from using them to classify video sequences might be informative for classifying environmental time-lapse images like those from PhenoCam cameras [54].

A computer-human hybrid system is one way to potentially achieve very high accuracy with a small amount of additional human effort. Images would be initially run through the trained CNN and both their predictions (“snow” or “no snow”) and their confidence measures would be calculated. For predictions below a particular confidence threshold, the associated image would be sent for human evaluation (either crowdsourced or expert) for an authoritative classification. Incorporating additional data about place, time, and the classifications of images before and after the target image into the decision about whether to send an image to a human or not could boost accuracy and efficiency even further. Such a hybrid system, called CrowdSearch, was developed to enable mobile phone users to perform image searches. In this system, the goal was to find similar images from the Internet matching a target image taken by the camera of a mobile phone. By using machine learning to identify candidate matches and then human participants for validation of those images, CrowdSearch performed at over 95% precision in near-real time [55].

### 3. MODIS validation

The accuracy of the MODIS snow product validated against our crowdsourced classifications (93.6%) and the CNNs (88.7% to 93.8%) fell in the middle of the wide range of reported validation studies (67%-99%) [44]. Our sensitivity for the MODIS snow product was 76.6%, somewhat lower than an evaluation of 11 years of the same product conducted for Colorado and Washington (78%-88%) [44]. Most of the MODIS errors are in missing small amounts of snow; there are few false positives. Given that MODIS pixels cover an area of 500m x 500m, determining whether snow is present in small amounts is a sub-pixel problem that has received attention for more than a decade [56–58] and continues to be a challenge [59]. It is recognized that errors may increase in the MODIS product when there is less than 20% snow cover [44]. Trees were not a problem for MODIS detection of snow if snow was present on the trees themselves, but the rate of “missed snow” was high when snow was present on the ground, but not on the trees. Previous studies have noted that the accuracy of the MODIS snow product is typically lower for densely forested areas [44–46].

**Fig 4:**
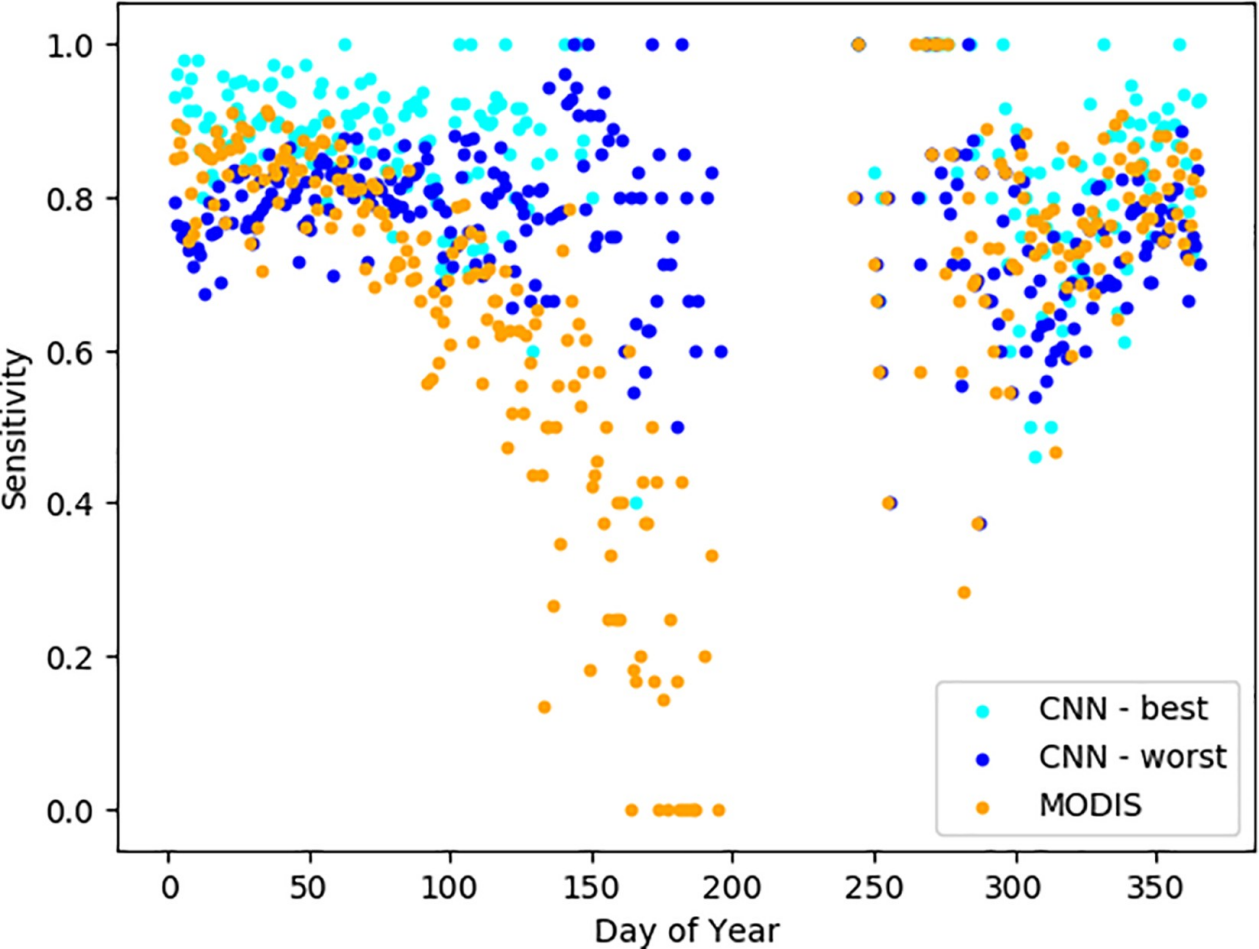
Sensitivity (true positive rate) by time of year for MODIS data and for two trained deep convolutional neural network (CNNs), as validated by the crowdsourced dataset. Data are not shown for days of year for which there were fewer than five images from which to determine the sensitivity.

The sensitivity for MODIS snow detection was highest in the winter, with slightly lower sensitivity in the fall with high variation and a precipitous decline in snow detection in the spring (Fig 4). Other studies have noted similar patterns, suggesting that the decreased detection of snow in spring is due to increased snow patchiness, snow being obscured by new vegetation, or the sensitivity of the MODIS snow fractional cover algorithm to land surface temperature [44,60]. Except in the spring, the sensitivity for MODIS fell in the range of the sensitivity for the CNNs. However, in the spring, even the worst-performing CNN maintained a reasonably high sensitivity, albeit with high variability, whereas the MODIS sensitivity declined. By contrast, the sensitivity for CNNs remains relatively high during the spring, though there is a lot of variation. While images with small amounts of snow are difficult to classify for both CNNs and for MODIS snow algorithms, they two approaches had trouble with different images. As a result, an approach in which we combine MODIS and CNN classifications yields much better results, boosting the MODIS sensitivity from 77% to ~90%. For the remaining ~10% of images, both methods miss the presence of snow.

**Fig 5:**
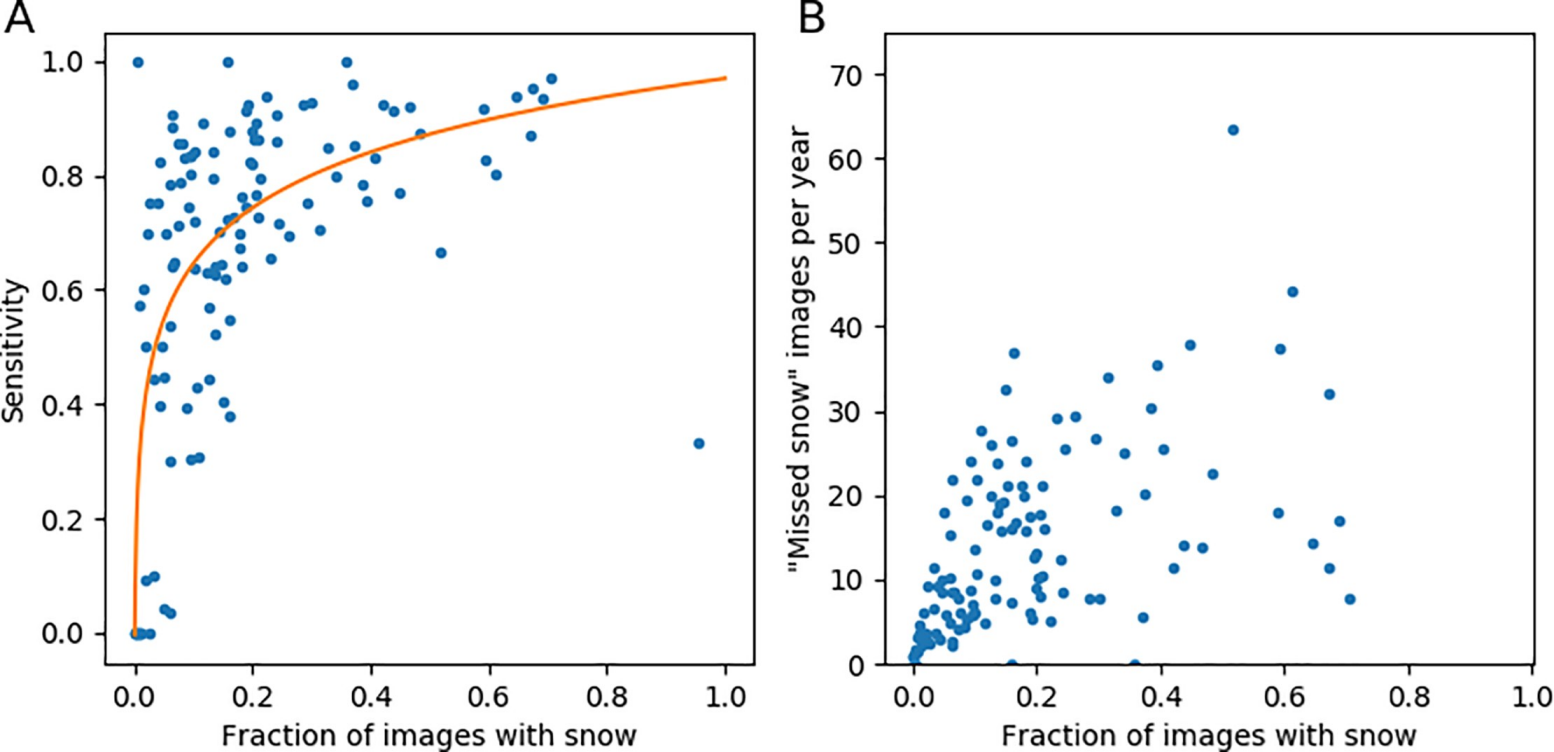
Sites with more snow have a higher MODIS sensitivity than those with less snow. They also have more images per year in which MODIS misses the presence of snow. (a) Sites with more snow tend to have a higher MODIS sensitivity. The fit logarithmic function explains 43% of variation. The outlier is the site at Port Alsworth, Lake Clark National Park and Preserve, Alaska. (b) Sites with more snow tend to have a greater number of images for which MODIS misses the snow. The Port Alsworth outlier is not shown; MODIS missed snow at this site 14 times out of 21 images, which scales to a “missed snow” rate of 232 days per year. The camera at Port Alsworth has known color balance issues.

**Fig 6:**
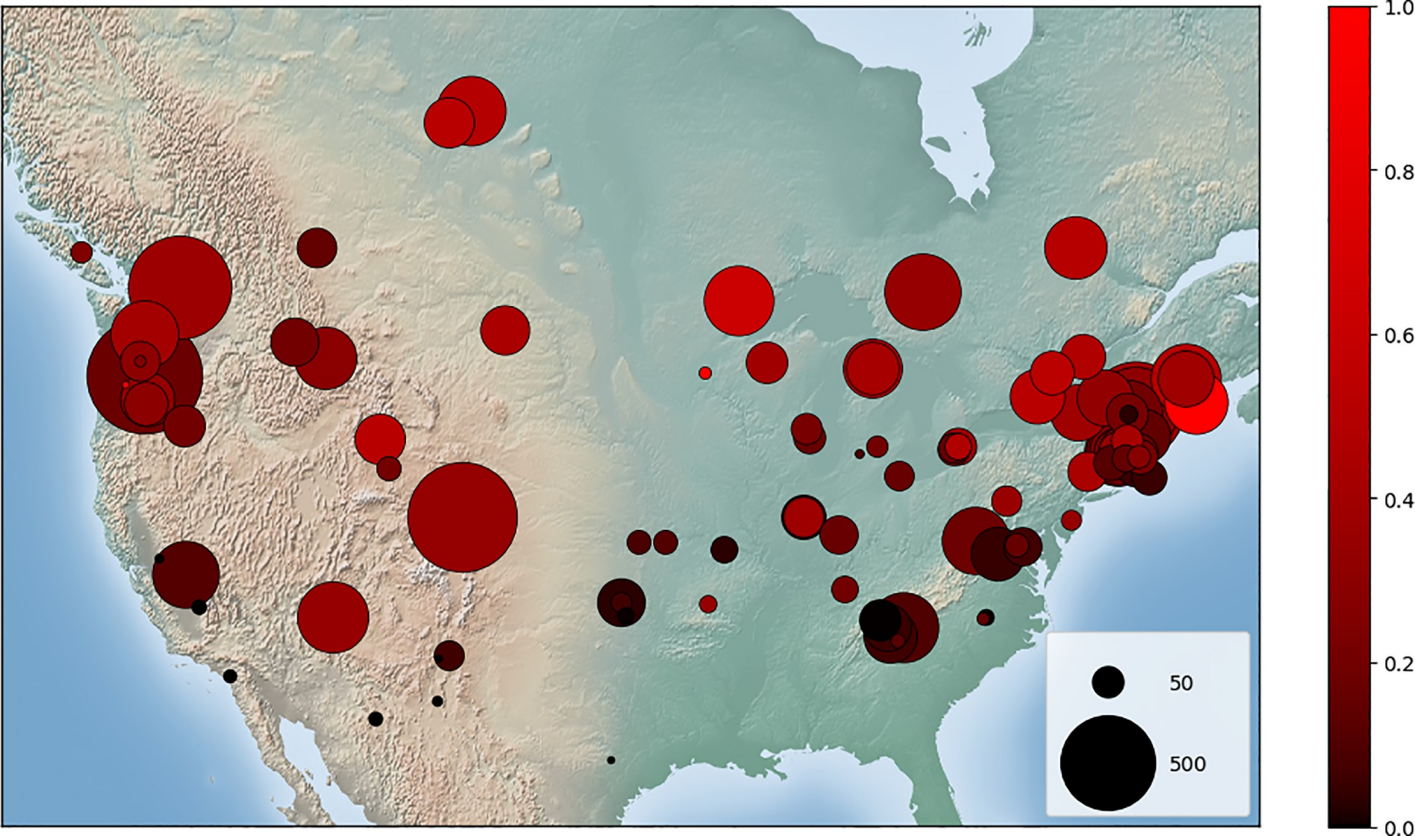
MODIS sensitivity for camera sites. Circle color indicates the sensitivity for each site. The size of each circle indicates the number of images containing snow, according to the crowdsourced data. Made with Natural Earth: free vector and raster map data @ naturalearthdata.com.

We investigated whether there was a geographic pattern in MODIS accuracy among sites. Sites with a higher fraction of snow days tended to have a higher accuracy and sensitivity than sites with less snow (Fig 5A), but there is a lot of variability and a logarithmic function fit to the data explains only 43% of the variation. Only sites with < 20% snow days have a sensitivity less than 50%, but there are also many sites with < 20% snow days that have high sensitivity. The sites with many snow days (> 30% snow days) have relatively high sensitivity, rarely lower than 70% (Fig 5A). We thought that this pattern might be explained by the greater number of patchy snow days relative to days of complete snow cover at sites with less snow. However, we did not find this to be the case. For sites with little snow, the number of “missed snow” errors was relatively small, as there were few snow days overall, whereas for sites with a lot of snow, the number of “missed snow” errors could be quite large even if the overall true positive rate was low (Fig 5B). Because amount of snow was correlated with latitude, we found that higher latitude sites had higher sensitivity on average (Fig 6).

## Conclusion

We have demonstrated an automated method for creating location-specific, high temporal frequency, data on the presence or absence of snow using imagery from automated, near-surface camera monitoring sites. Though we use only about a hundred cameras that were originally set up for a different purpose, such a method shows potential for improving snow monitoring. While the individual cameras cover only a small spatial extent, they were selected to sample across a wide range of North American ecosystems and human land use. As a result, we expect the model to perform well generally for images of temperate North American outdoor scenes, and especially for those that were created using the PhenoCam camera protocol. The cameras are also necessarily limited in number, but the strength of their high accuracy and hourly images can be leveraged by combining camera data with data from technologies that cover high spatial extents, such as satellite and aerial imagery, and possibly crowdsourced image databases such as Flickr.

The uses for our dataset of labeled images are numerous. They could be used to validate MODIS snow products and to help refine their algorithmic approaches. Additionally, the dataset could be used as input to snow models that use data streams from satellites or airborne platforms or crowdsourced image databases to create more accurate continuous predictions of snow. For example, MODIS snow data could be used to create a model of snow extent and then data from surface-based cameras could be used to refine model parameters for higher accuracy or to fill in gaps inferentially when clouds prevent direct measurement. Because our data processing system is automated, such models could be run in near real time. Computer vision researchers might also use the variety of high-quality outdoor images in our labeled dataset as a baseline to better capture information from crowdsourced outdoor image datasets that are noisier in nature.

We expect that our general method of automated sensors, crowdsourced labels, and deep learning for data processing will be implementable for a broad range of complex environmental datasets. We have shown that this method can achieve up to 98% accuracy for automated detection of snow from stationary time-lapse cameras. Other types of automated imagery–from animal camera traps, from remote sensors on satellites and airborne platforms, and from deep sea mapping cameras, for example–can also benefit from this approach. Deep learning has also been shown to be effective in processing audio [61] and video [62] streams, and so audio environmental data, such as animal acoustics, as well as video environmental data, such as animal behavior video monitoring, can potentially benefit from this method as well. The potential of our method for real-time large-scale acquisition and processing of ecological and environmental data opens up new research questions and provides increased information for natural resource managers.

## Supporting information

S1 Table PhenoCam sites used in the analysis and their characteristics. ‘Number of Images’ refers to the number of images used at each site that did not have a crowd consensus label of ‘bad image’.
(CSV)

S2 Table Filenames for the 172,927 good images used in the analyses and their crowd consensus labels. See [33] to access the original images.
(CSV)

S3 Table Filenames for the 2013 images with gold standard classifications. See [33] to access the original images.
(CSV)

S4 Table SVM output weights for determining snow vs. no snow from the last (fc7) layer of the trained Places365-VGG model. (CSV)

S1 Appendix Confusion matrices for crowdsource consensus, CNN-SVM models, and MODIS data product. (PDF)

S1 Fig View from the PhenoCam at Paradise Jackson Visitor Center, Mount Rainier National Park, Washington, USA. (TIF)
