# Supplementary material for: Integrating camera imagery, crowdsourcing, and deep learning to improve high-frequency automated monitoring of snow at continental-to-global scales
Source: PLoS One. 2018 Dec 27;13(12):e0209649. doi: 10.1371/journal.pone.0209649 (PMC6307743; doi:10.1371/journal.pone.0209649)
Supplement: S1 Appendix — (PDF) [file pone.0209649.s005.pdf]

Confusion matrices

		Expert Gold Standard	
		Snow	No Snow
Crowd	Snow	453	11
	No Snow	5	1328

		Crowd	
		Snow	No Snow
CNN-SVM with 10-fold cross validation, fully random; Type I, II, III sites	Snow	34,572	778
	No Snow	4,155	133,422

		Crowd	
		Snow	No Snow
CNN-SVM with 10-fold cross validation, fully random; Type I, II sites only	Snow	25,937	477
	No Snow	2,775	97,719

		Crowd	
		Snow	No Snow
CNN-SVM with 10-fold cross validation, fully random; Type I sites only	Snow	17,463	247
	No Snow	1,834	63,154

		Crowd	
		Snow	No Snow
CNN-SVM with 10-fold cross validation by site; Type I, II, III sites	Snow	32,261	8,050
	No Snow	6,466	126,150

		Crowd	
		Snow	No Snow
CNN-SVM with 10-fold cross validation by site; Type I, II sites only	Snow	23,973	3,275
	No Snow	4,739	94,921

		Crowd	
		Snow	No Snow
CNN-SVM with 10-fold cross validation by site; Type I sites only	Snow	16,592	2,666
	No Snow	2,705	60,735

		Crowd	
		Snow	No Snow
Places365-VGG using top-5 categories; Type I, II, III sites	Snow	22,695	12,998
	No Snow	16,032	121,202

		Crowd	
		Snow	No Snow
Places365-VGG using top-5 categories; Type I, II sites only	Snow	14,425	3,766
	No Snow	14,287	94,430

Places365-VGG using top-5 categories; Type I sites only

	Crowd: Snow	Crowd: No Snow
Snow	9,617	2,340
No Snow	9,680	61,061

		Expert Gold Standard	
		Snow	No Snow
MODIS	Snow	143	15
	No Snow	32	854

		Crowd	
		Snow	No Snow
MODIS	Snow	11,089	2,802
	No Snow	3,381	78,879

		Best CNN-SVM (Type I sites only, fully randomized)	
		Snow	No Snow
MODIS	Snow	4,969	1,911
	No Snow	850	36,780
