# Supplementary figures and images for: Integrating camera imagery, crowdsourcing, and deep learning to improve high-frequency automated monitoring of snow at continental-to-global scales
Source: PLoS One. 2018 Dec 27;13(12):e0209649. doi: 10.1371/journal.pone.0209649 (PMC6307743; doi:10.1371/journal.pone.0209649)

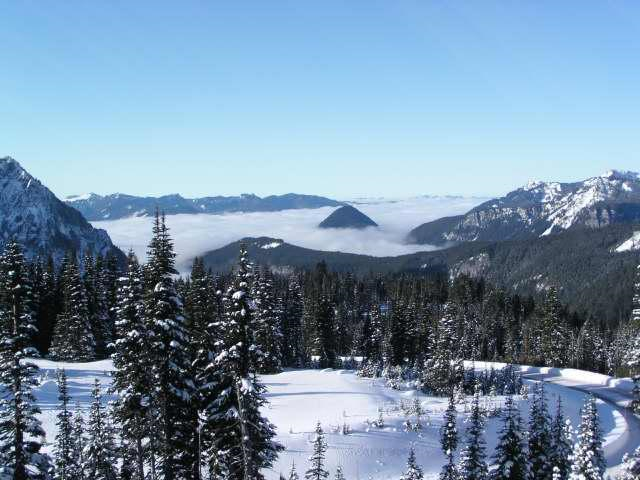

Supplement: S1 Fig — (TIF) [file pone.0209649.s006.tif]
